# Anti-tumor necrosis factor therapy in patients with inflammatory bowel disease; comorbidity, not patient age, is a predictor of severe adverse events

**DOI:** 10.1007/s00384-020-03716-6

**Published:** 2020-08-28

**Authors:** Vera E. R. Asscher, Quirine van der Vliet, Karen van der Aalst, Anniek van der Aalst, Eelco C. Brand, Andrea E. van der Meulen-de Jong, Bas Oldenburg, Marieke J. Pierik, Bas van Tuyl, Nofel Mahmmod, P. W. Jeroen Maljaars, Herma H. Fidder, On behalf of the Dutch ICC

**Affiliations:** 1 Department of Gastroenterology and Hepatology, Leiden University Medical Centre, Albinusdreef 2, 2333 ZA Leiden, the Netherlands; 2 Department of Gastroenterology and Hepatology, University Medical Centre Utrecht, Utrecht, the Netherlands; 3 Department of Gastroenterology and Hepatology, Maastricht University Medical Centre, Maastricht, the Netherlands; 4 Department of Gastroenterology and Hepatology, Diakonessenhuis Utrecht, Utrecht, the Netherlands; 5 Department of Gastroenterology and Hepatology, Sint Antonius Hospital Nieuwegein, Nieuwegein, the Netherlands

**Keywords:** Crohn’s disease, Ulcerative colitis, Biologicals, Elderly, Co-morbidity

## Abstract

**Purpose:**

To assess safety and effectiveness of anti-tumor necrosis factor (anti-TNF) therapy in IBD patients ≥ 60 years.

**Methods:**

Ninety IBD patients ≥ 60 years at initiation of anti-TNF therapy, 145 IBD patients ≥ 60 years without anti-TNF therapy and 257 IBD patients < 60 years at initiation of anti-TNF therapy were retrospectively included in this multicentre study. Primary outcome was the occurrence of severe adverse events (SAEs), serious infections and malignancies. Secondary outcome was effectiveness of therapy. Cox regression analyses were used to assess differences in safety and effectiveness. In safety analyses, first older patients with and without anti-TNF therapy and then older and younger patients with anti-TNF therapy were assessed.

**Results:**

In older IBD patients, the use of anti-TNF therapy was associated with serious infections (aHR 3.920, 95% CI 1.185–12.973, *p* = .025). In anti-TNF-exposed patients, cardiovascular disease associated with serious infections (aHR 3.279, 95% CI 1.098–9.790, *p* = .033) and the presence of multiple comorbidities (aHR 9.138 (1.248–66.935), *p* = .029) with malignancies, while patient age did not associate with safety outcomes. Effectiveness of therapy was not affected by age or comorbidity.

**Conclusion:**

Older patients receiving anti-TNF therapy have a higher risk of serious infections compared with older IBD patients without anti-TNF therapy, but not compared with younger patients receiving anti-TNF therapy. However, in anti-TNF-exposed patients, comorbidity was found to be an indicator with regards to SAEs. Effectiveness was comparable between older and younger patients.

**Electronic supplementary material:**

The online version of this article (10.1007/s00384-020-03716-6) contains supplementary material, which is available to authorized users.

## Introduction

As a consequence of the aging population and the rising prevalence of inflammatory bowel diseases (IBD), the group of older patients with Crohn’s disease (CD) or ulcerative colitis (UC) is enlarging. [1] Currently, approximately 10–30% of IBD patients is > 60 years old and about 10–15% of new IBD cases is diagnosed in patients > 60 years of age. [2–4]

Safety and effectiveness of medication may differ between older and younger patients, as a consequence of comorbidity, polypharmacy, senescence of the immune system or altered clearance of drugs. [5, 6] Results from clinical trials cannot be extrapolated to the older patient population with IBD because these patients are generally excluded from trial participation and available data from observational studies on the occurrence of severe adverse events (SAEs) in older IBD patients exposed to anti-TNF therapy are inconsistent. [7–10] Besides this, previous literature has been focussing on patient age, rather than comorbidity as a predictor of safety and effectiveness in patients with IBD receiving anti-tumor necrosis factor (TNF) therapy.

The aim of the present study is therefore to assess safety and effectiveness of anti-TNF therapy in patients with IBD aged 60 years and older while accounting for the presence of comorbidities.

## Methods

### Patients

This is a retrospective multicentre cohort study combining data from five hospitals in the Netherlands (University Medical Centre Utrecht (UMCU), St. Antonius Hospital Nieuwegein, Diakonessenhuis Utrecht, Leiden University Medical Centre (LUMC) and Maastricht University Medical Centre (MUMC)) on the effect of patient age on safety and effectiveness of anti-TNF therapy (infliximab (IFX), adalimumab (ADA) or certolizumab pegol (CZP)). Ethical approval has been granted by the medical research ethics committee Leiden The Hague Delft, MREC registration number G20.057 and research was conducted in accordance with the ethical standards as laid down in the declaration of Helsinki. Patients with an established IBD diagnosis were assigned to one of the three groups.[11] First, patients exposed to anti-TNF therapy for the first time at ≥ 60 years, second, patients without any exposure to anti-TNF therapy aged ≥ 60 years and third, patients < 60 years exposed to anti-TNF therapy.

The following data were collected: age, sex, diagnosis, date of diagnosis, duration of follow-up, number of IBD-related hospitalizations and data on anti-TNF therapy and immunosuppressive medication. Oral prednisone treatment was documented if prescribed for a period of at least 6 months at daily doses of ≥ 7.5 mg. Hepatic comorbidities (steatosis, drug-induced liver disease, chronic hepatitis B, chronic hepatitis C and alcoholic liver disease), gastro-intestinal comorbidities (celiac disease, diverticular disease, ischemic colitis, drug-induced colitis and radiation enteropathy), cardiovascular comorbidities (acute mesenteric ischemia, ischemic heart disease, cerebrovascular disease and hypertension), pulmonary comorbidities (asthmatic bronchitis and chronic obstructive pulmonary disease) and the presence of diabetes were recorded.

### Outcomes

Primary outcome was safety, defined as the occurrence of any SAE, serious infection or malignancy. Any SAE was defined as any event that resulted in (prolonging) hospitalization, was fatal or life-threatening or led to significant disability. Serious infections were defined similarly. A malignancy was considered a SAE. In addition, serious infections and malignancies were analysed separately as safety outcomes. Hospitalization at start of anti-TNF therapy was not considered a SAE.

Secondary outcome was effectiveness of therapy, defined by duration of anti-TNF therapy and treatment response, which was categorized as total sustained clinical benefit (primary clinical benefit or secondary clinical benefit, see below) or no sustained clinical benefit. Patients still receiving anti-TNF therapy at the last day of follow-up or in whom therapy had been discontinued because of remission were assigned to the total sustained clinical benefit group. If anti-TNF therapy had never been switched to another type of TNF inhibitor, total sustained clinical benefit was scored as primary clinical benefit. Total sustained clinical benefit after one or more switches of anti-TNF therapy was labelled secondary clinical benefit. If anti-TNF therapy was discontinued because of primary non-response, loss of response, occurrence of an adverse event or any other reason, patients were classified as having ‘no sustained clinical benefit’. Primary non-response was defined as lack of improvement of clinical signs and symptoms after induction therapy. Loss of response was defined as recurrence of disease activity during maintenance therapy after achieving an appropriate induction response. [12]

### Statistical analysis

All analyses were performed using IBM SPSS Statistics version 23.0 (SPSS, Inc, Chicago, IL). For continuous data, descriptive statistics were calculated as means with standard deviations (SD) when data were normally distributed and medians with interquartile ranges (IQR) when not normally distributed. Comparisons between groups were performed using independent sample *t* test or Mann-Whitney *U* test. Categorical variables were reported using absolute numbers and percentages, comparisons were performed using the *χ*^2^ test or Fisher’s exact test. Cox proportional hazards model was used to assess the effect of patient age and comorbidity on primary and secondary outcomes and to assess the effect of therapy as a time-dependent variable on primary outcome. To assess whether comorbidity or patient age at start of first anti-TNF therapy affected effectiveness outcomes, Cox proportional hazards model was used. Safety was defined in three different outcomes: any SAE, serious infection and malignancy. For any SAE and malignancy analyses, events occurring until end of follow-up were used. For serious infections, events occurring up until 3 months after last administration of medication were used.

To assess the influence of anti-TNF therapy on the occurrence of infections and malignancies as a time-dependent variable, exposure to anti-TNF therapy was tested as a time-dependent covariate in a Cox proportional hazards model. Exposure time was defined as time from the first anti-TNF infusion until the occurrence of a SAE or end of follow-up. We selected older IBD patients with and without anti-TNF therapy and used duration of follow-up since date of diagnosis until end of follow-up as ‘time’ in the malignancy analysis and follow-up since date of diagnosis until 3 months after the last date of first anti-TNF therapy or end of follow-up in the infection analysis. Hospitalization of any infection or the diagnosis of any malignancy was used as ‘status’. In the analyses regarding malignancies, the covariates age, comorbidity (categorized in no comorbidity, one comorbidity and two or more comorbidities, or specified in cardiovascular disease and diabetes), use of immunosuppressive therapy (oral prednisone and MTX/thiopurine use), use of budesonide and use of anti-TNF therapy were used. In the analysis regarding serious infections, immunosuppressive therapy was not used as a covariate because a cut-off follow-up duration was applied and start and stop dates of immunosuppressive therapy had not been consistently documented. A *p* value of < .05 was considered statistically significant.

## Results

### Study population characteristics

We identified 347 IBD patients currently using anti-TNF therapy, of whom 90 were 60 years or older at initiation of anti-TNF therapy and of whom 257 patients were younger than 60 years at the start of anti-TNF therapy. An additional 145 anti-TNF naive IBD patients of 60 years or older served as controls. The first group of patients was included in the hospitals UMCU (24.4%), St. Antonius Hospital Nieuwegein (16.7%), Diakonessenhuis Utrecht (5.6%), LUMC (17.8%) and MUMC (35.7%). Patients from group 2 were included in the hospitals UMCU (81.7%), Sint Antonius Hospital Nieuwegein (1.9%), Diakonessenhuis Utrecht (4.7%), LUMC (4.3%) and MUMC (7.4%). Patients in group 3 were included in UMCU (99.3%) and LUMC (0.7%). Characteristics of these patients are shown in Table 1.

**Table 1:** Characteristics of study population

	Older patients with anti-TNF therapy (*n* = 90)	Older patients without anti-TNF therapy (*n* = 145)	Younger patients with anti-TNF therapy (*n* = 257)
Age at inclusion in years, median [IQR]	68.72 [66.74–73.00]	69.44 [65.01–75.02]	37.43 [27.72–20.24]***
Male, *n* (%)	49 (54.4)	84 (57.9)	121 (47.1)
Age at diagnosis, mean (±SD)	52.15 (16.10)	46.39 (15.75)**	26.16 (11.27)***
Age at start anti-TNF therapy, mean (±SD)	67.56 (6.00)	n.a.	34.18 (12.94)***
Disease duration in years, median [IQR]	16.67 [5.73–29.48]	21.31 [12.04–34.14]**	10.14 [5.58–18.23]*
Duration of FU in weeks (start first anti-TNF till end FU or stop therapy) median [IQR]	70.50 [34.00–155.25]	n.a.	110.00 [41.50–217.00]*
Duration of FU in months (date diagnosis till malignancy or end FU), median [IQR]	194.00 [66.00–322.50]	249.00 [144.00–396.00]	n.a.
Duration of total anti-TNF therapy in years, median [IQR]	1.72 [0.81–4.04]	n.a.	3.34 [1.44–5.41]***
Type of IBD, *n* (%)			
CD	56 (62.2)	67 (46.2)**	206 (80.2)**
UC	30 (33.3)	71 (49.0)**	44 (17.1)**
IBD-U/IC	4 (4.4)	7 (4.8)	7 (2.7)
Montreal classification, *n* (%)			
CD location L1/L2/L3/L4	11 (19.6)/18 (32.1)/26 (46.2)/ 1 (1.8)	14 (21.2)/25 (37.9)/27 (40.9)/0 (0.0)	30 (14.6)/61 (29.6)/112 (54.4)/3 (1.5)
CD behaviour B1/B2/B3	19 (34.5)/26 (47.3)/10 (18.2)	36 (53.7)/17 (25.4)/14 (20.9)*	81 (39.5)/68 (33.2)/56 (27.3)
Perianal disease	21 (41.1)	10 (15.2)**	85 (43.4)
UC extension E1/E2/E3	0 (0.0)/14 (45.2)/17 (54.8)	2 (2.9)/29 (42.6)/37(54.4)	1 (2.2)/12 (26.7)/32 (71.1)
Comorbidity, *n* (%)			
Hepatic	6 (6.7)	14 (9.7)	8 (3.1)
Gastrointestinal	19 (21.1)	28 (19.3)	6 (2.3)***
Cardiovascular	35 (38.9)	74 (51.0)	34 (13.2)***
Pulmonary	10 (11.1)	18 (12.4)	18 (7.0)
Diabetes mellitus	14 (15.7)	18 (12.4)	12 (4.7)**
Comorbidity, *n* (%)			
No comorbidity	35 (38.9)	47 (32.4)	191 (74.3)***
One comorbidity	33 (36.7)	55 (37.9)	54 (21.0)
Two or more comorbidities	22 (24.4)	43 (29.7)	12 (4.7)
Type of TNF-inhibitor, *n* (%)			
IFX	67	n.a.	220*
ADA	44		129
CZP	0 (0.0)		5
Immunosuppressant, *n* (%)			
Thiopurines/MTX	79 (87.8)	51 (35.4)***	247 (96.1)**

Older inflammatory bowel disease (IBD) patients (≥ 60 years) at initiation of anti-tumor necrosis factor (anti-TNF) therapy were compared with older IBD patients (≥ 60 years) without any anti-TNF therapy and with younger IBD patients aged < 60 years at initiation of anti-TNF therapy. Significant differences are shown. ***p < .001, **p < .01, *p < .05

*IQR* interquartile range, *SD* standard deviation, *FU* follow-up, *CD* Crohn’s disease, *UC* ulcerative colitis, *IBD-U* IBD unclassified, *IC* indeterminate colitis, *IFX* infliximab, *ADA* adalimumab, *CZP* certoluzimab pegol, *MTX* methotrexate

L4 was reported when only upper gastrointestinal (GI) disease was present, CD location was missing in *N* = 1 (group 3), CD behaviour was missing in *N* = 1 (group 1) and *N* = 1 (group 2), perianal disease was missing in *N* = 5 (group 1), *N* = 10 (group 2) and *N* = 1 (group 3), UC extension was missing in *N* = 3 (group 1), *N* = 6 (group 2) and *N* = 10 (group 3). The use of MTX was missing in *N* = 1 in the group with older IBD patients without anti-TNF therapy

Older patients receiving anti-TNF therapy less often had CD compared with patients receiving anti-TNF therapy at a younger age. The older patients more often had diabetes, gastro-intestinal, cardiovascular and other comorbidities and less often used MTX or thiopurine therapy compared with younger patients with anti-TNF therapy (87.8% versus 96.1%, *p* < .010). Patients in the anti-TNF naive group were diagnosed at a younger age when compared with the older patients on anti-TNF therapy. Anti-TNF naive patients differed from the older patients on anti-TNF therapy with respect to diagnosis (less CD, more UC) and the use of immunosuppressive therapy (less thiopurines or MTX). Comorbidity rates were similar between older IBD patients with and without anti-TNF therapy.

### Does anti-TNF therapy influence the occurrence of safety outcomes in older patients?

To assess the effect of anti-TNF therapy on safety outcomes in older patients, all older patients were analysed using date of diagnosis as start of follow-up, with the use of anti-TNF therapy as a time-dependent covariate.

Twenty-eight serious infections occurred during follow-up. Use of anti-TNF therapy was found to increase risk of serious infections (aHR 3.920, 95% CI 1.185–12.973, *p* = .025) in multivariable Cox regression. Age at diagnosis, number of comorbidities (Table 2) and the presence of cardiovascular disease or diabetes (data not shown) did not affect the risk of serious infections.

**Table 2:** Univariable and multivariable analysis on the occurrence of serious infections in older anti-TNF users and older non-users, using follow-up time from date of diagnosis until 3 months after last administration of anti-TNF therapy or end of follow-up

	Univariable analysis			Multivariable analysis		
	HR	95% CI	*P*	HR	95% CI	*P*
Anti-TNF therapy	5.131	1.679–15.684	0.004	3.920	1.185–12.973	0.025
Age at diagnosis	1.026	0.997–1.057	0.078	1.018	0.987–1.049	0.264
Comorbidity^a^						
1	1.308	0.550–3.112	0.544	1.164	0.487–2.785	0.733
2 or more	1.202	0.445–3.247	0.717	0.999	0.363–2.751	0.998

*HR* hazard ratio, *CI* confidence interval, *TNF* tumor necrosis factor

^a^Reference is zero comorbidities

Twenty-six malignancies occurred during follow-up. Univariable and multivariable Cox regression analysis did not show an association between the use of anti-TNF therapy and the development of malignancies during follow-up (Table 3). The presence of the comorbidities diabetes or cardiovascular disease was also not associated with occurrence of malignancies (data not shown). Infections and malignancies are presented in supplementary Tables 1 and 2.

**Table 3:** Univariable and multivariable analysis on the occurrence of malignancies in older anti-TNF users and older non-users, using follow-up time from date of diagnosis until end of follow-up

	Univariable analysis			Multivariable analysis		
	HR	95% CI	*P*	HR	95% CI	*P*
Anti-TNF therapy	2.617	0.770–8.894	0.123	1.422	0.284–7.128	0.668
Age at diagnosis	1.046	1.010–1.082	0.011	1.025	0.989–1.063	0.178
Comorbidity^a^						
1	3.248	1.058–9.971	0.040	2.706	0.844–8.683	0.094
2 or more	4.036	1.238–13.156	0.021	2.869	0.809–10.183	0.103
Budesonide	1.681	0.771–3.663	0.191	1.746	0.758–4.018	0.190
Oral prednisone	0.721	0.287–1.815	0.488	1.012	0.365–2.802	0.982
MTX and/or thiopurine use	0.700	0.321–1.527	0.270	0.630	0.245–1.621	0.338

*HR* hazard ratio, *CI* confidence interval, *TNF* tumor necrosis factor, *MTX* methotrexate

^a^Reference is zero comorbidities

### Do patient age or comorbidity affect the safety of anti-TNF therapy

To assess the effect of patient age and comorbidity on safety outcomes in anti-TNF therapy, all anti-TNF-exposed patients were analysed. One hundred and eighteen SAEs (any SAE) occurred after start of first anti-TNF therapy, the majority (46, 40.0%) because of exacerbation of disease and 26 (22.3%) because of IBD-related surgery. Age at start of anti-TNF therapy and comorbidity were not associated with the occurrence of any SAE (supplementary Table 3a). The incidence of IBD-related surgery did not differ between older and younger anti-TNF users (5 out of 90 patients (5.6%) versus 21 out of 257 (8.2%), *p* = 0.417).

Twenty serious infections occurred after start of anti-TNF therapy, but age did not affect the risk of occurrence. The presence of cardiovascular disease was independently associated with the occurrence of serious infections (aHR 3.279, 95% CI 1.098–9.790, *p* = .033), whereas presence of diabetes or the presence of any comorbidity was not (supplementary Table 3b).

Eight malignancies were diagnosed after start of anti-TNF therapy, age was not a risk factor (supplementary Tables 3c). The presence of two or more comorbidities was independently associated with the risk of developing a malignancy (aHR 9.138, 95% CI 1.248–66.935, *p* = .029, supplementary Table 3c). A list of all SAEs occurring after start of first anti-TNF therapy is presented in supplementary Tables 4, 5 and 6.

### Do patient age or comorbidity affect the effectiveness of anti-TNF therapy?

The clinical effectiveness of anti-TNF therapy did not differ between older and younger anti-TNF users (Table 4) or between patients with and without comorbidity. Follow up, defined as the time from first anti-TNF administration until discontinuation of therapy, was significantly shorter in the group of older patients compared with younger patients (median duration 70.5 weeks (34.0–155.3) versus 110.0 weeks (41.5–217.0), *p* = .017, Table 4). Follow-up did not differ between patients with and without comorbidity (median duration 114.0 weeks (47.0–221.0) versus 92.0 weeks (35.0–200.5), *p* = .161). When using Cox regression analysis, age at start of anti-TNF therapy, comorbidity and type of anti-TNF therapy did not affect duration of treatment (Table 5). The presence of diabetes or cardiovascular disease during follow-up did not significantly affect the duration of treatment as well (data not shown).

**Table 4:** Treatment response in older and younger anti-TNF users

	Older anti-TNF patients (*n* = 90)	Younger anti-TNF patients (*n* = 257)	*P* value
First anti-TNF therapy, *n* (%)			
IFX	59 (65.6)	199 (77.4)	0.035
ADA	31 (34.4)	58 (22.6)	
First anti-TNF treatment duration in weeks, median [IQR]	70.50 [34.00–155.25]	110.00 [41.50–217.00]	0.017
Stop date of first anti-TNF therapy *n* (%)			0.004
Year < 2005	0 (0,0)	14 (11.0)	
Year 2005–2009	6 (17.1)	46 (36.2)	
Year ≥ 2010	29 (82.9)	67 (52.8)	
Total sustained clinical benefit	73 (81.1)	201 (78.2)	0.653
Primary clinical benefit	55 (61.1)	133 (51.8)	0.141
Secondary clinical benefit	18 (20.0)	68 (26.5)	0.257
Stop reasons for first anti-TNF treatment, *n* (%)			
Primary non-responder	1 (2.9)	5 (4.7)	1.000
Secondary loss of response	9 (26.5)	72 (59.5)	0.001
Adverse event	19 (55.9)	34 (28.1)	0.004
Other	5 (14.7)	10 (8.3)	0.323

Treatment response compared between patients on anti-TNF therapy aged ≥ 60 years and aged < 60 years. Stop date of first anti-TNF therapy: the year in which the first anti-TNF therapy was stopped. Total sustained clinical benefit: still receiving anti-TNF therapy at last day of FU or anti-TNF therapy discontinuation because of remission. Primary clinical benefit: no switch to other anti-TNF therapy during FU and still receiving anti-TNF therapy at last day of FU or discontinuation of remission of disease. Secondary clinical benefit: clinical benefit after one or more switches of anti-TNF therapy. Primary non-responder: lack of improvement of clinical signs and symptoms after induction therapy. Secondary loss of response: recurrence of disease activity during maintenance therapy after achieving an appropriate induction response. Percentages per stop reason were calculated as percentage of all stop reasons per group

*TNF* tumor necrosis factor, *IQR* interquartile range, *IFX* infliximab, *ADA* adalimumab, *FU* follow-up

**Table 5:** Univariable and multivariable analysis on duration of first anti-TNF treatment

	Univariable analysis			Multivariable analysis		
	HR	95% CI	*P*	HR	95% CI	*P*
Age at start therapy	0.999	0.991–1.008	0.906	1.000	0.990–1.010	0.966
Comorbidity^a^						
1	0.739	0.501–1.088	0.126	0.731	0.484–1.104	0.137
2 or more	1.130	0.676–1.887	0.642	1.130	0.635–2.012	0.678
Type of anti-TNF therapy^b^	1.087	0.753–1.568	0.657	1.114	0.767–1.620	0.570

*HR* hazard ratio, *CI* confidence interval, *TNF* tumor necrosis factor

^a^Reference is zero comorbidities

^b^Reference is infliximab therapy (certolizumab pegol was not used as first anti-TNF therapy)

The number of older patients discontinuing anti-TNF therapy because of adverse events was significantly higher compared with younger patients (55.9% versus 28.1%, *p* = .003, Table 4) while the number of older patients stopping because of loss of response was significantly lower (26.5% versus 59.5%, *p* = .001, Table 4). The number of patients with comorbidity discontinuing anti-TNF therapy because of adverse events was significantly higher compared with patients without comorbidity (47.1% versus 27.9%, *p* = .018). The number of patients with comorbidity stopping because of loss of response was lower as compared with patients without comorbidity, although this did not differ significantly (43.1% versus 56.7%, *p* = .111). Reasons for stopping are presented in supplementary Table 7.

## Discussion

In this multicentre study, we found that the presence of comorbidity was a better indicator of serious infections and malignancies in anti-TNF-exposed patients than patient age. Effectiveness of anti-TNF therapy was comparable between older and younger patients. Reasons for cessation of therapy did differ, being more often due to adverse events in older patients and patients with comorbidity.

In our study, exposure to anti-TNF treatment increased the risk of serious infections in older patients with IBD. In the Treat registry, an increase in serious infections was observed in anti-TNF-treated IBD patients. [13] Lobaton et al. found a higher incidence of serious infections in patients aged ≥ 65 years on anti-TNF therapy as compared with older patients using immunosuppressive medication and/or corticosteroids. [8] More recently, two meta-analyses were published assessing safety risks of biologics in older patients with IBD. Both Piovani et al. (RR 2.70, 95% CI 1.56–4.66, serious infections) and Borren and Ananthakrishnan (OR 11.22; 95% CI 3.60–34.99, any infection) found that the risk of infections was substantially increased when comparing older anti-TNF users to older non-users.[14, 15]

When comparing younger to older patients, we used age at start of follow-up as a factor in multivariate analysis instead of using an arbitrary cut-off at 60 or 65 years of age. As ageing is a gradual process with a steady reduction of physiologic reserves, this strategy may be a better way to evaluate the role of ageing on occurrence of serious infections and malignancies. In our study, patient age did not affect the occurrence of SAEs, serious infections and malignancies. However, presence of cardiovascular disease did increase the risk of serious infections, and the presence of multiple comorbidities increased the risk of developing a malignancy. These findings are in contrast to those of the Borren and Ananthakrishnan meta-analysis, in which older patients had a higher risk of malignancy (OR, 3.47; 95% CI, 1.71–7.03) and infection (OR, 3.48; 95% CI, 1.98–6.14). This may be due to the fact that the patients in a number of these studies were older [16, 17] and may have had more comorbidities. Especially this latter factor may be important as studies on toxicity of chemotherapy found that comorbidity increases the risk for toxicity and is a better indicator for toxicity risk than patient age.[16, 17] Previous studies found that presence of comorbidities increased the risk for adverse events in response to immunosuppressive treatments. [18, 19]

Desai et al. concluded that older IBD patients were less likely to respond to anti-TNF therapy and had a shorter drug survival as compared with younger patients. Among both patient groups, comorbidity (Charlson Comorbidity Index (CCI) > 0) was associated with anti-TNF therapy discontinuation. This could be due to polypharmacy combined with altered drug absorption, distribution and elimination [20] or due to an increased opportunity for drug interactions because of polypharmacy for multiple morbidities. [21] In line with a recent study on persistence of anti-TNF therapy in older patients with IBD by Porcari et al. [22], we observed a shorter treatment duration in older patients compared with younger patients. However, when analysing both patient groups together using Cox regression analysis and correcting for confounders, age at start of therapy did not affect treatment duration. Older patients did discontinue therapy more frequently due to adverse events, and less often due to loss of response, as compared with younger patients. This has also been described by Desai et al.[7] Both our results and the study by Porcari et al. did not find comorbidity to affect time to cessation of anti-TNF therapy while Desai et al. found increasing comorbidity to be associated with treatment cessation.[7] Regarding effectiveness, Lobaton et al. only found a reduced short-term response to anti-TNF therapy in older patients, but this difference disappeared after 6 months. [8]

Our study has some limitations, in addition to those inherent to any study with a retrospective design. Clinical activity scores were not available, as a result of which data on clinical treatment response were based on comments in medical reports instead of disease activity scores. Comorbidity scores were based on the sum of comorbidities because information to calculate a comorbidity score such as the CCI was not fully available. However, because data in all patients were obtained from medical reports, reporting bias would have affected all patients equally. Furthermore, younger patients exposed to anti-TNF therapy were mostly (93.4%) included in referral centres and the older non-anti-TNF users were included in referral hospitals only. One could argue that this could have affected comparability of patients, although we assume that in all hospitals, international guidelines considering anti-TNF therapy are maintained, especially concerning reasons to stop therapy. The older non-anti-TNF users, although included in a referral centre, had a milder disease compared with older anti-TNF users, as expressed by the infrequent use of immunomodulatory medication in this group during follow-up.

The strength of our study lies in the large number of patients included and the multicentre aspect; patients were included from three referral hospitals and two general hospitals. We believe that our study therefore provides reliable and generalizable data on the effect of anti-TNF compounds in older patients with IBD.

In conclusion, this study shows that the presence of comorbidities, and not an increasing age, is a risk factor for SAEs in IBD patients on anti-TNF therapy. Older patients receiving anti-TNF therapy have a higher risk of serious infections compared with older IBD patients without anti-TNF therapy, but not compared with younger IBD patients receiving anti-TNF therapy. Effectiveness of therapy was comparable between older and younger patients but older patients tend to stop therapy more often because of adverse events and less often due to loss of response compared with younger patients. Careful monitoring of the older IBD patient with multiple comorbidities receiving anti-TNF therapy is recommended.

## Electronic supplementary material

ESM 1 (DOCX 44 kb).
