# Supplementary material for: Anti-tumor necrosis factor therapy in patients with inflammatory bowel disease; comorbidity, not patient age, is a predictor of severe adverse events
Source: Int J Colorectal Dis. 2020 Aug 28;35(12):2331–8. doi: 10.1007/s00384-020-03716-6 (PMC7648742; doi:10.1007/s00384-020-03716-6)
Supplement: Supplementary file 1 — (DOCX 44 kb). [file 384_2020_3716_MOESM1_ESM.docx]

**Supplementary table 1. List of malignancies occurring during follow-up from date of diagnosis in older anti-TNF users and older non-users**

| Patient ID | Age at start follow-up | Time after diagnosis in months | Event type |
| --- | --- | --- | --- |
| 441 | 16,13 | 404 | Colorectal carcinoma |
| 363 | 16,96 | 486 | Colorectal cancer |
| 347 | 20,26 | 433 | Breast cancer |
| 16 | 23,81 | 156 | Pleomorphic adenoma of the parotid gland |
| 442 | 24,18 | 502 | Prostate cancer |
| 416 | 28,96 | 345 | Urothelial carcinoma |
| 448 | 29,28 | 285 | Colorectal carcinoma |
| 17 | 30,33 | 324 | Prostate cancer |
| 48 | 31,81 | 322 | Prostate cancer |
| 444 | 32,64 | 244 | Endometrial cancer |
| 428 | 37,19 | 434 | Non-Hodgkin lymphoma |
| 326 | 37,75 | 233 | Prostate cancer |
| 440 | 38,41 | 300 | Colorectal carcinoma |
| 325 | 42,33 | 248 | Pancreatic carcinoma |
| 360 | 43,04 | 288 | Colorectal cancer |
| 382 | 43,49 | 208 | Prostate cancer |
| 402 | 44,42 | 271 | Oesophageal carcinoma |
| 61 | 46,8 | 254 | Breast cancer |
| 438 | 51,63 | 313 | Colorectal carcinoma |
| 6 | 52,29 | 162 | Pancreatic carcinoma |
| 394 | 52,65 | 176 | Prostate cancer |
| 310 | 56,22 | 24 | Lung cancer |
| 348 | 61,89 | 0 | Colorectal cancer |
| 72 | 62,71 | 32 | Prostate cancer |
| 322 | 67,85 | 96 | Breast cancer |
| 53 | 70,82 | 97 | Renal cell cancer |

**Supplementary table 2. List of infections occurring after date of IBD diagnosis until 3 months after last administration of anti-TNF therapy or until end of follow up in older users and older non-users**

| Patient ID | Age at start follow-up | Time after diagnosis in months | Event type |
| --- | --- | --- | --- |
| 1 | 25,80 | 156 | Perianal abscess |
| 17 | 30,33 | 235 | Fever |
| 26 | 59,93 | 3 | Perianal abscess |
| 28 | 46,58 | 153 | Drainage of abscess |
| 29 | 18,34 | 577 | Fever due to bacterial translocation |
| 32 | 24,75 | 114 | Incision abdominal abscess |
| 42 | 37,19 | 292 | Drainage abdominal abscess |
| 43 | 30,70 | 455 | Cholangitis (suspect) |
| 44 | 42,75 | 229 | Perianal abscess next to pouch |
| 47 | 46,60 | 6 | Drainage perianal abscesses |
| 52 | 31,13 | 317 | CMV pouchitis and ileitis |
| 58 | 29,63 | 264 | Drainage of abscess |
| 70 | 29,99 | 124 | Incision and drainage perianal abscess |
| 71 | 71,35 | 0 | Fever and cold shivers |
| 323 | 20,47 | 264 | Abdominal abscess |
| 341 | 53,15 | 82 | Abscess right lower abdomen |
| 365 | 43,38 | 335 | Septic abdomen |
| 375 | 66,38 | 14 | Abscess |
| 381 | 54,11 | 138 | Abscess |
| 382 | 43,49 | 35 | Abscess |
| 388 | 43,03 | 124 | Incision and drainage perianal abscess |
| 395 | 56,54 | 230 | Intraabdominal abscess |
| 402 | 44,42 | 119 | Abdominal pain and fever |
| 410 | 50,48 | 13 | Gastroenteritis |
| 412 | 53,87 | 13 | Abscess |
| 451 | 42,00 | 110 | Douglas abscess |
| 480 | 70,24 | 50 | Abscess |
| 491 | 64,68 | 2 | Herpes zoster infection |

| **Supplementary table 3a. Univariable and multivariable analysis on the occurrence of any SAE in older and younger IBD patients on anti-TNF therapy** | | | | | | |
| --- | --- | --- | --- | --- | --- | --- |
|  | Univariable Analysis |  |  | Multivariable Analysis |  |  |
|  | HR | 95% CI | P | HR | 95% CI | P |
| Age at start therapy | 0.988 | 0.988-1.008 | 0.629 | 0.992 | 0.980-1.003 | 0.154 |
| Diabetes | 1.740 | 0.977-3.098 | 0.060 | 1.783 | 0.990-3.211 | 0.054 |
| Cardiovascular Disease | 1.311 | 0.853-2.016 | 0.217 | 1.503 | 0.912-2.477 | 0.110 |

|  | Univariable Analysis |  |  | Multivariable Analysis |  |  |
| --- | --- | --- | --- | --- | --- | --- |
|  | HR | 95% CI | P | HR | 95% CI | P |
| Age at start therapy | 0.988 | 0.988-1.008 | 0.629 | 0.994 | 0.983-1.006 | 0.313 |
| Comorbidity* 1 2 or more | 0.961 1.435 | 0.620-1.491 0.811-2.541 | 0.860 0.215 | 1.042 1.678 | 0.655-1.658 0.879-3.206 | 0.862 0.117 |
| HR=Hazard Ratio, CI=Confidence Interval *reference is zero comorbidities | | | | | | |

| **Supplementary table 3b. Univariable and multivariable analysis on the occurrence of serious infections in older and younger IBD patients on anti-TNF therapy** | | | | | | |
| --- | --- | --- | --- | --- | --- | --- |
|  | Univariable Analysis |  |  | Multivariable Analysis |  |  |
|  | HR | 95% CI | P | HR | 95% CI | P |
| Age at start therapy | 1.008 | 0.984-1.033 | 0.513 | 1.002 | 0.975-1.029 | 0.907 |
| Comorbidity* 1 2 or more | 2.004 1.552 | 0.790-5.087 0.336-7.166 | 0.144 0.573 | 1.962 1.492 | 0.724-5.318 0.282-7.893 | 0.185 0.638 |
| HR=Hazard Ratio, CI=Confidence Interval *reference is zero comorbidities | | | | | | |

|  | | | | | | |
| --- | --- | --- | --- | --- | --- | --- |
|  | Univariable Analysis |  |  | Multivariable Analysis |  |  |
|  | HR | 95% CI | P | HR | 95% CI | P |
| Age at start therapy | 1.008 | 0.984-1.033 | 0.513 | 0.992 | 0.964-1.020 | 0.559 |
| Diabetes | 1.992 | 0.572-6.938 | 0.279 | 1.684 | 0.473-6.000 | 0.421 |
| Cardiovascular disease | 2.882 | 1.1727.085 | 0.021 | 3.279 | 1.098-9.790 | 0.033 |

| **Supplementary table 3c. Univariable and multivariable analysis on the occurrence of malignancies in older and younger IBD patients on anti-TNF therapy** | | | | | | |
| --- | --- | --- | --- | --- | --- | --- |
|  | Univariable Analysis |  |  | Multivariable Analysis |  |  |
|  | HR | 95% CI | P | HR | 95% CI | P |
| Age at start therapy | 1.061 | 1.015-1.109 | 0.009 | 1.050 | 0.995-1.107 | 0.076 |
| Diabetes | 6.506 | 1.535-27.571 | 0.011 | 3.970 | 0.929-16.961 | 0.063 |
| Cardiovascular disease | 4.250 | 1.059-17.049 | 0.041 | 1.593 | 0.338-7.519 | 0.556 |

|  | Univariable Analysis |  |  | Multivariable Analysis |  |  |
| --- | --- | --- | --- | --- | --- | --- |
|  | HR | 95% CI | P | HR | 95% CI | P |
| Age at start therapy | 1.061 | 1.015-1.109 | 0.009 | 1.035 | 0.979-1.095 | 0.223 |
| Comorbidity* 1 2 or more | 1.406 19.545 | 0.127-15.534 3.741-102.101 | 0.781 0.000 | 0.837 9.138 | 0.065-10.734 1.248-66.935 | 0.892 0.029 |
| HR=Hazard Ratio, CI=Confidence Interval *reference is zero comorbidities | | | | | | |

**Supplementary table 4. List of any serious adverse events after start of anti-TNF therapy up until end of follow-up**

| Patient ID | Age at start anti-TNF therapy in years | Time after start therapy in weeks | SAE category | SAE |
| --- | --- | --- | --- | --- |
| 281 | 11,4 | 14 | Exacerbation | Exacerbation |
| 306 | 12,31 | 382 | Exacerbation | Exacerbation |
| 243 | 14,23 | 3 | Exacerbation | Exacerbation |
| 222 | 15,27 | 49 | IBD Surgery | Ileocecal resection |
| 187 | 15,73 | 15 | Exacerbation | Exacerbation |
| 213 | 16,11 | 215 | Exacerbation | Exacerbation |
| 97 | 17,37 | 6 | Exacerbation | Exacerbation |
| 221 | 17,73 | 23 | IBD related complication or symptom | Hematoma, abscess right lower abdomen and perihepatic abscess |
| 207 | 18,13 | 80 | Exacerbation | Exacerbation |
| 147 | 18,62 | 28 | IBD Surgery | Ileocecal resection |
| 287 | 18,66 | 23 | IBD related complication or symptom | Abdominal pain |
| 194 | 18,71 | 316 | Exacerbation | Exacerbation |
| 120 | 18,86 | 144 | Exacerbation | Exacerbation |
| 226 | 19,09 | 132 | Exacerbation | Exacerbation |
| 98 | 20,11 | 89 | IBD Surgery | Subtotal colectomy |
| 85 | 20,56 | 169 | Exacerbation/ Infection | Exacerbation or urinary tract infection |
| 160 | 20,91 | 399 | IBD Surgery | Subtotal colectomy |
| 138 | 21,1 | 213 | Medication complication | Sensibility loss of the lower legs |
| 181 | 22,35 | 180 | IBD related complication | Ileocecal abscess |
| 174 | 22,38 | 329 | Exacerbation | Exacerbation |
| 252 | 22,6 | 38 | IBD Surgery | Ileocecal resection |
| 275 | 22,86 | 41 | IBD related complication or symptom | Perianal abscess |
| 141 | 22,9 | 135 | IBD related complication or symptom | Ileitis |
| 122 | 23,02 | 38 | IBD related complication or symptom | Therapy refractory disease |
| 105 | 23,29 | 34 | Exacerbation | Exacerbation |
| 121 | 23,56 | 113 | IBD related complication or symptom | Extended perianal fistulae |
| 90 | 23,88 | 107 | Exacerbation | Exacerbation |
| 305 | 24,04 | 36 | IBD related complication or symptom | Pyoderma gangrenosum |
| 268 | 24,09 | 66 | IBD Surgery | Proctocolectomy |
| 175 | 24,36 | 24 | Exacerbation | Exacerbation |
| 303 | 24,58 | 46 | Exacerbation | Exacerbation |
| 162 | 25,22 | 4 | Exacerbation | Exacerbation |
| 215 | 25,61 | 223 | IBD related complication or symptom | Rectal blood loss |
| 307 | 26,26 | 37 | IBD Surgery | Ileocecal resection |
| 135 | 26,86 | 208 | IBD Surgery | Proctocolectomy |
| 134 | 26,89 | 8 | Exacerbation | Exacerbation |
| 152 | 27 | 177 | IBD Surgery | Subtotal colectomy |
| 113 | 27,71 | 51 | IBD related complication or symptom | Protein losing enteropathy |
| 273 | 27,77 | 147 | IBD related complication or symptom | Abdominal pain |
| 262 | 28,34 | 250 | IBD related complication or symptom | Electrolyte disorder |
| 237 | 28,71 | 152 | Exacerbation | Exacerbation |
| 115 | 28,9 | 375 | IBD related complication or symptom | Diarrhoea and vomiting |
| 286 | 29,13 | 45 | Exacerbation | Exacerbation |
| 283 | 29,4 | 14 | Exacerbation | Exacerbation |
| 119 | 29,46 | 290 | Exacerbation | Exacerbation |
| 225 | 29,68 | 19 | Exacerbation | Exacerbation |
| 255 | 30,1 | 79 | IBD related complication or symptom | Stenosis lineal flexure |
| 117 | 30,18 | 45 | IBD Surgery | Ileocecal resection |
| 278 | 31,16 | 140 | IBD related complication or symptom | Ileus |
| 267 | 31,45 | 34 | Exacerbation | Exacerbation |
| 302 | 33,51 | 121 | Exacerbation | Exacerbation |
| 233 | 33,67 | 64 | Infection | Viral enteritis |
| 288 | 34,18 | 19 | Exacerbation | Exacerbation |
| 197 | 34,97 | 72 | Exacerbation | Exacerbation |
| 201 | 36,13 | 5 | IBD related complication or symptom | Stenosis ileum |
| 171 | 36,38 | 1 | IBD Surgery | Right hemicolectomy |
| 92 | 36,4 | 64 | IBD Surgery | Ileocecal resection |
| 308 | 37,25 | 321 | Other | Clinical evaluation of Crohn’s disease after six weeks treatment with oral prednisone |
| 304 | 37,86 | 106 | IBD Surgery | Part of jejunum resection |
| 294 | 38,15 | 105 | Infection | Bacterial meningitis |
| 139 | 38,31 | 19 | IBD Surgery | Part of jejunum resection |
| 195 | 39,55 | 184 | Exacerbation | Exacerbation |
| 188 | 39,94 | 22 | Exacerbation | Exacerbation |
| 227 | 39,98 | 43 | Other | Clinical injection adalimumab because of previous allergic reaction |
| 190 | 40,12 | 208 | Exacerbation | Exacerbation |
| 251 | 40,22 | 148 | Exacerbation | Exacerbation |
| 154 | 40,54 | 39 | IBD Surgery | Ileocecal resection |
| 186 | 40,82 | 270 | IBD related complication or symptom | Clinical colonoscopy because of dysplasia and crohn’s disease |
| 108 | 41,56 | 108 | IBD Surgery | Subtotal colectomy |
| 127 | 42,7 | 12 | Exacerbation | Exacerbation |
| 89 | 43,48 | 69 | IBD related complication or symptom | Pancreatitis and primary sclerosing cholangitis |
| 191 | 43,99 | 42 | IBD Surgery | Right hemicolectomy |
| 232 | 44,17 | 368 | Exacerbation | Exacerbation |
| 78 | 44,66 | 328 | Malignancy | Urothelial cell carcinoma |
| 218 | 44,69 | 28 | Exacerbation | Exacerbation |
| 246 | 44,81 | 3 | IBD related complication or symptom | Constipation |
| 256 | 47,08 | 153 | Malignancy | Breast cancer |
| 295 | 47,48 | 82 | Infection | CMV infection |
| 276 | 47,7 | 193 | IBD Surgery | Ileocecal resection |
| 236 | 48,02 | 144 | IBD related complication or symptom | Abdominal pain |
| 462 | 49,83 | 4 | IBD related complication or symptom | Stenosis |
| 457 | 51,26 | 544 | IBD related complication or symptom/ Infection | Fever and perianal abscess |
| 456 | 51,55 | 1 | IBD Surgery | Incision and drainage of perianal abscess |
| 196 | 52,39 | 60 | Exacerbation | Exacerbation |
| 142 | 53,69 | 176 | Exacerbation | Exacerbation |
| 467 | 54,11 | 205 | IBD related complication or symptom | Perianal abscess |
| 470 | 54,22 | 9 | Medication complication | Nausea and vomiting during azathiopurine treatment |
| 460 | 54,75 | 6 | IBD related complication or symptom | Anemia |
| 466 | 56,6 | 213 | IBD related complication or symptom | Ileus |
| 472 | 56,77 | 28 | IBD Surgery | Colectomy |
| 478 | 57,46 | 177 | Infection | Fever |
| 82 | 58,43 | 4 | IBD Surgery | Subtotal colectomy |
| 487 | 59,46 | 6 | Exacerbation | Exacerbation |
| 54 | 60,76 | 11 | IBD Surgery | Subtotal colectomy |
| 6 | 61,63 | 43 | IBD Surgery | Subtotal colectomy |
| 70 | 61,74 | 10 | Exacerbation | Exacerbation |
| 44 | 61,77 | 6 | IBD related complication or symptom | Perianal abscess |
| 29 | 62,13 | 207 | IBD related complication or symptom | Stenosis ileum |
| 19 | 62,93 | 176 | Exacerbation | Exacerbation |
| 41 | 64,12 | 50 | IBD related complication or symptom | Ileus |
| 38 | 64,16 | 41 | Exacerbation | Exacerbation |
| 15 | 64,57 | 6 | IBD Surgery | Subtotal colectomy |
| 7 | 64,64 | 80 | IBD Surgery | Subtotal colectomy |
| 32 | 64,83 | 15 | Infection | Cryptosporidium infection |
| 40 | 65,14 | 77 | IBD related complication or symptom | Ileus |
| 11 | 65,66 | 41 | Exacerbation | Exacerbation |
| 64 | 65,71 | 68 | IBD related complication or symptom | Constipation |
| 476 | 65,77 | 24 | IBD Surgery | Sigmoid resection |
| 33 | 66,21 | 254 | IBD related complication or symptom | Rectal blood loss and anemia |
| 43 | 67,18 | 10 | IBD related complication or symptom | Stenosis |
| 51 | 67,59 | 42 | Exacerbation | Exacerbation |
| 26 | 68,4 | 65 | Infection | Pneumonia |
| 62 | 69,52 | 77 | Infection | Listeria bacteraemia |
| 14 | 71,73 | 46 | Exacerbation | Exacerbation |
| 28 | 72,58 | 10 | Exacerbation | Exacerbation |
| 12 | 73,94 | 262 | Exacerbation | Exacerbation |
| 10 | 74,09 | 1 | Exacerbation | Exacerbation |
| 53 | 76,85 | 108 | Malignancy | Renal cell tumor |

**Supplementary table 5. List of serious infections after start of anti-TNF therapy until 12 weeks after last administration of anti-TNF medication**

| Patient ID | Age at start anti-TNF therapy in years | Time after start therapy in weeks | Infection |
| --- | --- | --- | --- |
| 221 | 17,73 | 23 | Perihepatic and right lower abdomen abscess |
| 85 | 20,56 | 169 | Exacerbation or urinary tract infection |
| 252 | 22,6 | 19 | Cecal abscess |
| 275 | 22,86 | 41 | Perianal abscess |
| 159 | 24,2 | 15 | Drainage of abscess |
| 303 | 24,58 | 250 | Drainage of abscess |
| 233 | 33,67 | 64 | Viral enteritis |
| 92 | 36,4 | 11 | Drainage of abscess |
| 294 | 38,15 | 105 | Bacterial meningitis |
| 251 | 40,22 | 519 | Perianal abscess |
| 295 | 47,48 | 82 | CMV infection |
| 457 | 51,26 | 544 | Perianal abscess |
| 456 | 51,55 | 1 | Incision and drainage perianal abscess |
| 467 | 54,11 | 205 | Perianal abscess |
| 472 | 56,77 | 196 | Gastro-enteritis |
| 478 | 57,46 | 181 | Fever |
| 487 | 59,46 | 457 | Pneumonia |
| 44 | 61,77 | 6 | Perianal abscess |
| 32 | 64,83 | 15 | Cryptosporidium infection |
| 43 | 67,18 | 78 | Cholangitis |

**Supplementary table 6. List of malignancies after start of anti-TNF therapy until end of follow-up**

| Patient ID | Age at start anti-TNF therapy | Time after start therapy in weeks | Malignancy |
| --- | --- | --- | --- |
| 89 | 43,48 | 298 | Biliary adenocarcinoma |
| 78 | 44,66 | 328 | Urothelial carcinoma |
| 218 | 44,69 | 582 | Renal cell carcinoma |
| 256 | 47,08 | 153 | Breast cancer |
| 487 | 59,46 | 0 | Renal cell carcinoma |
| 6 | 61,63 | 221 | Pancreatic cancer |
| 61 | 65,83 | 113 | Breast cancer |
| 53 | 76,85 | 108 | Renal cell carcinoma |

**Supplementary table 7. List of stop reasons for first anti-TNF therapy**

| Patient ID | Age at start anti-TNF therapy | Time until stop therapy in weeks | Category | Stop reason |
| --- | --- | --- | --- | --- |
| 281 | 11,40 | 150 | Adverse event | Allergic reaction |
| 238 | 11,55 | 285 | Loss of response |  |
| 123 | 15,12 | 219 | Loss of response |  |
| 222 | 15,27 | 87 | Loss of response |  |
| 187 | 15,73 | 17 | Loss of response |  |
| 86 | 16,31 | 8 | Adverse event | None specified |
| 207 | 18,13 | 9 | Loss of response |  |
| 110 | 18,32 | 36 | Loss of response |  |
| 179 | 18,51 | 32 | Loss of response |  |
| 194 | 18,71 | 260 | Loss of response |  |
| 120 | 18,86 | 97 | Loss of response |  |
| 266 | 18,97 | 8 | Non response at first admission |  |
| 226 | 19,09 | 202 | Loss of response |  |
| 98 | 20,11 | 8 | Adverse event | Allergic reaction with antibodies |
| 160 | 20,91 | 133 | Adverse event | None specified |
| 138 | 21,1 | 160 | Loss of response |  |
| 203 | 21,35 | 297 | Adverse event | None specified |
| 249 | 21,64 | 69 | Adverse event | Alopecia |
| 177 | 21,73 | 17 | Loss of response |  |
| 157 | 21,86 | 25 | Loss of response |  |
| 100 | 22,15 | 53 | Other | Patients wish; tiredness after admissions |
| 181 | 22,35 | 26 | Loss of response |  |
| 174 | 22,38 | 331 | Loss of response | Loss of response |
| 275 | 22,86 | 43 | Loss of response |  |
| 141 | 22,9 | 164 | Other | Pregnancy wish |
| 296 | 22,91 | 8 | Non response at first admission |  |
| 122 | 23,02 | 43 | Loss of response |  |
| 137 | 23,02 | 2 | Non response at first admission |  |
| 81 | 23,09 | 104 | Adverse event | None specified |
| 105 | 23,29 | 70 | Other | Painful injection sites |
| 121 | 23,56 | 492 | Loss of response |  |
| 136 | 23,58 | 104 | Other | Logistic reasons |
| 90 | 23,88 | 47 | Loss of response |  |
| 305 | 24,04 | 14 | Loss of response |  |
| 268 | 24,09 | 13 | Adverse event | Allergic reaction |
| 159 | 24,2 | 56 | Loss of response |  |
| 175 | 24,36 | 25 | Adverse event | None specified |
| 303 | 24,58 | 243 | Loss of response |  |
| 146 | 25,01 | 16 | Adverse reaction | Allergic reaction with antibodies |
| 162 | 25,22 | 305 | Other | None specified |
| 104 | 25,43 | 69 | Loss of response |  |
| 215 | 25,61 | 192 | Other | Discontinuation at patients own initiative |
| 106 | 25,87 | 39 | Loss of response |  |
| 214 | 26,23 | 340 | Loss of response |  |
| 307 | 26,26 | 200 | Adverse event | Back pain |
| 135 | 26,86 | 76 | Loss of response |  |
| 134 | 26,89 | 13 | Loss of response |  |
| 152 | 27 | 10 | Adverse event | Non specified |
| 113 | 27,71 | 306 | Loss of response |  |
| 273 | 27,77 | 15 | Adverse event | Itching injection site |
| 242 | 28,73 | 2 | Loss of response |  |
| 115 | 28,9 | 180 | Loss of response |  |
| 291 | 29,08 | 50 | Loss of response |  |
| 286 | 29,13 | 0,57 | Adverse event | Infiltrate at site of injection |
| 165 | 29,35 | 690 | Adverse event | None specified |
| 283 | 29,4 | 47 | Loss of response |  |
| 119 | 29,46 | 70 | Other | Discontinuation at patients own initiative |
| 220 | 29,6 | 5 | Other | Surgical intervention |
| 225 | 29,68 | 4 | Other | No reason for treatment discontinuation found |
| 247 | 29,79 | 40 | Loss of response |  |
| 255 | 30,1 | 133 | Loss of response |  |
| 117 | 30,18 | 12 | Loss of response |  |
| 184 | 30,41 | 2 | Adverse event | None specified |
| 130 | 31,05 | 14 | Loss of response |  |
| 300 | 31,11 | 1 | Adverse event | Delayed hypersensitivity reaction |
| 278 | 31,16 | 39 | Adverse event | Allergic reaction |
| 267 | 31,45 | 26 | Loss of response |  |
| 211 | 32,61 | 156 | Loss of response |  |
| 166 | 32,76 | 140 | Loss of response |  |
| 302 | 33,51 | 4 | Non response at first admission |  |
| 87 | 33,71 | 63 | Loss of response |  |
| 197 | 34,97 | 131 | Adverse event | None specified |
| 308 | 37,25 | 6 | Non response at first admission |  |
| 253 | 37,47 | 26 | Loss of response |  |
| 99 | 37,48 | 115 | Loss of response |  |
| 167 | 37,68 | 189 | Loss of response |  |
| 304 | 37,86 | 49 | Adverse event | Hyperpigmentation |
| 299 | 38,01 | 184 | Loss of response |  |
| 173 | 38,24 | 20 | Loss of response |  |
| 176 | 39,05 | 6 | Loss of response |  |
| 114 | 39,48 | 52 | Loss of response |  |
| 188 | 39,94 | 86 | Loss of response |  |
| 227 | 39,98 | 1 | Adverse event | Allergic reaction |
| 190 | 40,12 | 95 | Adverse event | None specified |
| 251 | 40,22 | 513 | Loss of response |  |
| 124 | 40,31 | 52 | Loss of response |  |
| 154 | 40,54 | 39 | Loss of response |  |
| 186 | 40,82 | 52 | Loss of response |  |
| 289 | 41,16 | 6 | Adverse event | Skin reaction |
| 108 | 41,56 | 57 | Loss of response |  |
| 208 | 42,33 | 24 | Loss of response |  |
| 127 | 42,7 | 49 | Adverse event | None specified |
| 89 | 43,48 | 61 | Loss of response |  |
| 189 | 43,56 | 297 | Loss of response |  |
| 191 | 43,99 | 26 | Loss of response |  |
| 78 | 44,66 | 115 | Adverse event | Herpes simplex virus keratitis and patients wish to stop medication |
| 218 | 44,69 | 71 | Loss of response |  |
| 246 | 44,81 | 74 | Loss of response |  |
| 206 | 45,88 | 53 | Loss of response |  |
| 490 | 46,47 | 387 | Loss of response |  |
| 297 | 47,07 | 40 | Loss of response |  |
| 133 | 48,5 | 8 | Adverse event | Allergic reaction |
| 204 | 49,38 | 37 | Loss of response |  |
| 280 | 49,4 | 253 | Loss of response |  |
| 205 | 49,74 | 35 | Loss of response |  |
| 462 | 49,83 | 115 | Adverse event | Hypersensitivity reaction |
| 301 | 49,95 | 52 | Loss of response |  |
| 309 | 50,89 | 199 | Loss of response |  |
| 240 | 52,15 | 1 | Adverse event | Delayed hypersensitivity reaction |
| 185 | 53,04 | 15 | Adverse event | None specified |
| 142 | 53,69 | 26 | Other | Arthralgia |
| 470 | 54,22 | 0 | Adverse event | Fainting |
| 465 | 54,29 | 32 | Adverse event | Flue-like symptoms |
| 469 | 56,06 | 160 | Loss of response |  |
| 455 | 57,17 | 50 | Adverse event | Herpes zoster infection |
| 144 | 57,39 | 47 | Adverse event | Hypersensitivity reaction |
| 478 | 57,46 | 365 | Loss of response |  |
| 475 | 58,62 | 95 | Loss of response |  |
| 461 | 58,92 | 2 | Adverse event | Allergic reaction |
| 463 | 58,99 | 59 | Loss of response |  |
| 16 | 60,46 | 1 | Adverse event | Allergic reaction (skin reaction and itching) |
| 489 | 61,12 | 21 | Adverse event | Itching |
| 6 | 61,63 | 43 | Loss of response |  |
| 70 | 61,74 | 11 | Other | Subtotal colectomy |
| 1 | 61,8 | 6 | Adverse event | None specified |
| 29 | 62,13 | 213 | Loss of response |  |
| 17 | 62,61 | 100 | Adverse event | Pericarditis and kidney failure |
| 19 | 62,93 | 8 | Adverse event | Skin reaction, muscle and joint complaints |
| 18 | 63,2 | 12 | Adverse event | Itching |
| 56 | 63,55 | 123 | Loss of response |  |
| 41 | 64,12 | 65 | Adverse event | Antibodies |
| 38 | 64,16 | 47 | Loss of response |  |
| 7 | 64,64 | 81 | Loss of response |  |
| 40 | 65,14 | 63 | Adverse event | None specified |
| 11 | 65,66 | 21 | Loss of response |  |
| 64 | 65,71 | 72 | Other | No venous access |
| 50 | 65,79 | 104 | Adverse event | Other |
| 61 | 65,83 | 46 | Loss of response |  |
| 58 | 66,92 | 156 | Other | Stop reason not specified |
| 51 | 67,59 | 51 | Adverse event | Hypertension, dyspnoea, nausea |
| 31 | 67,89 | 35 | Adverse event | Muscle complaints |
| 26 | 68,4 | 43 | Adverse event | Skin reaction |
| 474 | 68,91 | 26 | Other | Wait gain, night sweating |
| 59 | 69,42 | 26 | Adverse event | Pneumonia |
| 62 | 69,52 | 0,14 | Adverse event | Listeria meningitis |
| 72 | 69,8 | 120 | Adverse event | Fever, cold shivers, hypotension |
| 12 | 73,94 | 52 | Adverse event | None specified |
| 10 | 74,09 | 1 | No response at first admission |  |
| 65 | 76,75 | 126 | Loss of response |  |
| 53 | 76,85 | 14 | Adverse event | Fever, cold shivers |
| 68 | 80,65 | 42 | Loss of response |  |
| 34 | 81,6 | 367 | Adverse event | None specified |
| 73 | 81,92 | 87 | Adverse event | Allergic reaction (dyspnoea) |
| 25 | 83,82 | 143 | Adverse event | Fever |
